# Incidence, Clinical Features, and Outcomes of the Confirmed Neonatal COVID-19 Infection in the Southwest Iran

**DOI:** 10.1155/2023/7095326

**Published:** 2023-09-27

**Authors:** Najmeh Maharlouei, Arash Khojasteh Zonoozi, Zaynab Noeizad, Atila Erami, Hamidreza Parsa, Zahra Eskandari Kootahi, Sara Raji, Kamran B. Lankarani

**Affiliations:** ^1^Community Medicine, Health Policy Research Center, Institute of Health, Shiraz University of Medical Sciences, Shiraz, Iran; ^2^Student Research Committee, School of Medicine, Mashhad University of Medical Sciences, Mashhad, Iran; ^3^Department of Child and Infant Health, Shiraz University of Medical Science, Shiraz, Iran; ^4^Persian Cohort Research Center, Mashhad University of Medical Sciences, Mashhad, Iran; ^5^Internal Medicine, Health Policy Research Center, Institute of Health, Shiraz University of Medical Sciences, Shiraz, Iran

## Abstract

**Background:**

The impact of COVID-19 on the neonatal population is still mysterious. This study is aimed at reporting the prevalence of COVID-19 and its clinical characteristics and outcomes among neonates in Iran.

**Methods:**

We conducted a retrospective cohort including 25 neonates who had COVID-19 infection confirmed by reverse transcription polymerase chain reaction (RT-PCR). Based on neonates' hospitalization records, data regarding neonatal and maternal characteristics and clinical and paraclinical findings were extracted.

**Results:**

In Fars Province, the incidence of COVID-19 among neonates was 47.5 per 100000 living births in one year. From 25 neonates, 20 cases (80%) were recovered, while five cases (20%) died, and all of them were symptomatic. Nine cases (37.5%) were preterm, and two cases (22.2%) belonged to deceased neonates. Four out of five deceased neonates (80%) suffered from congenital abnormalities, and all required respiratory support in the course of their disease progression. Also, 18 neonates (72%) were admitted to NICU. Moreover, the COVID-19 RT-PCR test of nine mothers (43.7%) became positive.

**Conclusions:**

This study showed that the incidence of confirmed and symptomatic SARS-CoV-2 infection among neonates in the Fars Province of Iran over one year was 47.5 per 100000 living births. Thoroughly evaluating the epidemiological factors associated with COVID-19, such as underlying health conditions and family history of COVID-19, is crucial in properly managing neonates during the pandemic and increasing awareness.

## 1. Introduction

With the emergence of the novel coronavirus disease 2019 (COVID-19) in December 2019, the world has been on a red alert experiencing a global health crisis resulting in the loss of many lives and major consequences on millions of others across the world [1]. Aside from the differences between previously known changes, which mainly appear with symptoms, such as fatigue, fever, dry cough, headache, and loss of sense of taste and smell, the virus can potentially infect all age groups. Although it significantly affects adults in older age ranges, the effects of acute respiratory coronavirus syndrome in children do not appear to be significant [2, 3]. The incidence of COVID-19 in children under 18 years of age is 2.1% [4]. A recent systematic review revealed that 50% of children under five years who were infected with COVID-19 were infants [5]. Another study showed a 6% pooled percentage of infected neonates with COVID-19 [6]. Although the current evidence shows a low risk of perinatal/postnatal COVID-19 infection, previous experiences of similar outbreaks, including respiratory syndrome coronavirus 1 (SARS-CoV-1) and Middle East respiratory syndrome coronavirus (MERS-CoV), have shown a relationship with poor pregnancy outcomes, which should be considered in future investigations [7, 8].

Neonates with COVID-19 are often asymptomatic or have mild symptoms, but severe respiratory distress syndrome can occur in those with underlying conditions [9]. The transmission of COVID-19 to neonates is not fully understood, but vertical intrauterine transmission or transmission via breastfeeding may be possible [5, 10]. Suspicion of infection in neonates is based on contact with an infected person, maternal infection around delivery [11], and common clinical findings such as dyspnea, fever, and cough [8, 9]. Studies have shown that neonates may require more respiratory support than older infants and children [12]. A recent review of Iranian pregnant women with COVID-19 found a mortality rate of 23.5% in neonates with confirmed infections [13].

Overall, there is still a paucity of information about the prevalence and outcomes of COVID-19 in neonates, both in Iran and in other countries, and most obtained data are related to some cases with a mild form of the disease, and many unknowns remain to be investigated [2, 13]. To rely on acceptable results extracted from local data in each region to facilitate decision-making processes and the implementation of macro policies, in this study, we investigated the incidence of COVID-19 infection among neonates in Fars Province, Iran, between March 20, 2020, and March 20, 2021.

## 2. Methods and Materials

### 2.1. Study Design and Participants

This retrospective cohort study investigated the records of 25 Iranian neonates born from March 21, 2020, to March 20, 2021, in Fars Province in Iran. Fars, the fourth populous province with 36 counties, located in the southwest of Iran, is one of the provinces with the most variety of ethnicities, including Fars, Lurs, Turks, Arabs, Kurds, Georgians, and Circassians. The study population consisted of all neonates born in Fars Province with COVID-19 infection confirmed via reverse transcription polymerase chain reaction (RT-PCR) test after the coronavirus infection was clinically suspected by the leading physician during hospitalization. Any cases born outside of Fars Province or with unavailable positive RT-PCR tests according to national guidelines were excluded from the study.

### 2.2. Data Collection

Census data were obtained from medical records through their hospitalization period in the respective hospitals. Investigators designed a standardized checklist to extract the target neonates' characteristics, clinical outcomes, and maternal risk factors. Neonatal characteristics, including birth height and weight, gender, APGAR scores at the 1st minute and the 5th minute after birth, gestational age at birth, multifetal pregnancy, blood group, feeding type, place of care, observed congenital anomalies, and living status at the end (alive versus deceased), were collected. Also, information about neonatal clinical outcomes, such as hospitalization length, the time between symptom onset to detection of COVID-19, age on admission, clinical symptoms, laboratory test results, close contact with a COVID-19 patient, positive family history, and receiving treatment were gathered. Maternal age, comorbidities, history of fever on admission, premature rupture of membranes (PROM), mode of delivery, RT-PCR test results, and isolation were collected as probable maternal risk factors.

### 2.3. Ethics Statement

To maintain patient confidentiality, the data are presented anonymously. The study protocol was performed following the Helsinki ethical principles, and the Ethics Committee of Shiraz University of Medical Sciences (SUMS) approved the study (IR.SUMS.REC.1399.1049).

### 2.4. Statistical Analysis

The data were analyzed via SPSS 26.0 (IBM Corp., Armonk, NY, USA). All categorical data were presented as frequencies and percentages, while all continuous variables were expressed as median, minimum, and maximum. The reduced sample size allowed only a simple description of the data. Comparisons between survived and deceased neonates as well as the analysis of risk factors were not possible.

## 3. Results

### 3.1. Study Design

The current study investigated 25 infants born between March 2020 and March 2021 with confirmed positive RT-PCR tests of COVID-19, recruited from hospitals affiliated with the SUMS. Based on the data released by the treatment vice-chancellor of SUMS, the total number of live births during the mentioned period was 52664. Hence, the incidence of RT-PCR-confirmed COVID-19 neonates from March 21, 2020, to March 20, 2021, was estimated to be 47.5 per 100000 living births.

### 3.2. Neonate Characteristics

The characteristics of the entire cohort can be found in Tables 1 and 2.

#### 3.2.1. Recovered Neonates

Based on the provided Tables 1 and 2, it can be concluded that the majority of recovered neonates had a birth weight ranging from 1490 to 3770 grams and a birth height ranging from 33 to 56 centimeters. Most of the neonates had a 1-minute APGAR score of 9 and a 5-minute APGAR score of 10. From all 15 males, 13 (86%) neonates recovered, and among 15 neonates with gestational age more than 37 weeks or later, 12 (80%) neonates were able to recover. Only two neonates were born from a multifetal pregnancy, and both recovered (100%). The blood type O was the most prevalent blood type among all neonates as well as the recovered neonates, accounting for 66.7% of cases, and all of them were Rh positive. The most common feeding type was breastfeeding (90.9%), followed by formula milk (66.7%) from all neonates. Six out of ten neonates with congenital anomalies were able to recover, with an admission mean postnatal age of 2 days (ranging from 0 to 25 days) to the hospital and the detection of COVID-19 symptoms occurring 2 days (ranging from 0 to 14 days) after the onset of symptoms. Recovered neonates had an average hospitalization duration of 10.5 days (ranging from 1 to 29 days). Fever and poor feeding were the most common clinical symptoms, each present in 50% of neonates, while cough and gastroenteritis were less frequent. Only one neonate had a positive result for C-reactive protein, but all neonates received antibiotic therapy. Additionally, 13 (52%) of all neonates had a family history of COVID-19, and 92.3% of them were able to recover. Among all neonates admitted to the NICU, 72.2% were able to recover, while 50% of neonates required mechanical ventilation during their hospitalization.

#### 3.2.2. Deceased Neonates

As shown in Tables 1 and 2, it can be concluded that out of a total of 25 neonates, 5 (20%) were deceased. The birth weight of deceased neonates ranged from 1490 to 3670 grams with an average of 2900 grams, and the birth height of deceased neonates ranged from 33 to 76 centimeters, with an average of 48 centimeters. The majority of neonates who died had a 1-minute APGAR score of 9 and a 5-minute APGAR score of 10. Out of 5 deceased neonates, 2 (40%) were female, and 3 (60%) were male. Among the deceased neonates, 3 (60%) were born before 37 weeks of gestational age, and 2 (40%) were born at 37 weeks of gestational age or later. None of the deceased neonates had blood type A or B. One neonate had a negative Rh factor, and the others had a positive Rh factor. The most common feeding type among deceased neonates was formula milk (33.3%), followed by breastfeeding (9.1%). Out of a total of 10 neonates with congenital anomalies or metabolic diseases, 4 (40%) were deceased, which is higher than the proportion of deceased neonates without congenital anomalies or metabolic diseases (6.7%). The average postnatal age at admission to the hospital for the deceased neonates was 12 days (ranging from 10 to 27 days). The duration between the onset of symptoms and the detection of COVID-19 was two days (ranging from 1 to 14 days), the same as the recovered neonates. The neonates who did not survive had an average hospitalization duration of 9 days (ranging from 3 to 19 days). Respiratory distress, lethargy, and poor feeding were observed in a significant proportion of the deceased neonates, with 4 (44.4%), 3 (25%), and 3 (23.1%) of them exhibiting these symptoms, respectively. Additionally, 1 (50%) of the deceased neonates had a positive result for C-reactive protein. Only 1 (11.1%) of the deceased neonates had a family history of COVID-19, and 3 (50%) required mechanical ventilation during their hospitalization. All deceased neonates had been admitted to NICU.

### 3.3. Maternal Characteristics

#### 3.3.1. Recovered Neonates

The study showed that all neonates born to mothers aged over 35 years or under 19. From nine neonates whom mothers tested positive for COVID-19 PCR, eight were recovered. Comorbid conditions in mothers were also noted, with diabetes mellitus (DM) being present in 2 (66.7%) mothers, chronic hypertension (HTN) in 1 (100%), hypothyroidism in 5 (83.4%), epilepsy in 4%, and addiction in 2 (100%). Maternal fever on admission was present in 80% of cases, and premature rupture of membranes was observed in 50% of cases. The majority of deliveries were via cesarean section (93.3%), with the most common indication being previous cesarean section or voluntary (100%). Finally, COVID-19 PCR testing was positive in 88.9% of cases, and 75% of mothers and their neonates were isolated from each other.

#### 3.3.2. Deceased Neonates

Table 3 shows the maternal characteristics as potential risk factors for neonatal COVID-19 infection. All of the parturients of deceased neonates aged between 19 and 35 years were regarded as low risk. Regarding comorbidities, diabetes, hypothyroidism, and epilepsy were reported in one mother separately. One mother (20%) was identified to have a fever on admission, and one of them had PROM as the adverse pregnancy outcome. About 40% of all mothers who had been delivered through vaginal delivery had deceased neonates. Positive RT-PCR tests were observed among one mother.

Moreover, based on the experts' opinions on cases, it appears that the source of COVID-19 is uncertain in nearly half of the reported cases (13, 54.2%), and the remaining cases are attributed to vertical transmission (3, 12.5%), family (5, 18.5%), or community (3, 12.5%) transmission.

### 3.4. Clinical Evolution of Deceased Neonates

**Figure 1:**
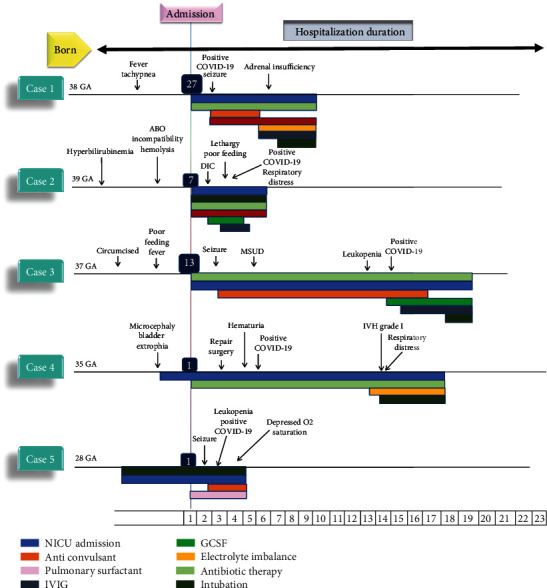
Summary timeline of five neonates prior to their death.

The summary timeline of five neonates prior to their death is illustrated in Figure 1. Pivotal clinical features related to the interpretation and findings of each deceased neonate are described concisely below.

#### 3.4.1. Case 1

A 27-day-old full-term neonate was admitted with fever, tachypnea, and irritability to rule out possible sepsis. He was delivered via home birth. Antibiotic therapy with ampicillin and cefotaxime was initiated at NICU. During hospitalization, she developed multiple seizure episodes, though her EEG and brain ultrasonography came normal. On the second day, the RT-PCR test result was positive, and isolation was implemented. Furthermore, she developed tachycardia (heart rate: 210 beats/minutes), and a cardiology consult was requested. The cardiologist performed echocardiography, which revealed mild pleural effusion and sinus tachycardia, and she was treated with adenosine. Due to hyperkalemia, hyponatremia, and acidosis, adrenal insufficiency was suspected. On the last days of hospitalization, the neonate also received intravenous immunoglobulin (IVIG) and was intubated due to severe respiratory distress. Regardless of support and resuscitation attempts, the neonate died.

#### 3.4.2. Case 2

A seven-day-old full-gestation neonate with a medical history of hyperbilirubinemia and jaundice due to ABO blood group incompatibility hemolysis had been admitted to NICU while she was intubated. At ten days of age, she developed lethargy and poor feeding, and the COVID-19 PCR sample was positive. On physical examination, rales were auscultated in the left lung, and a related chest X-ray (CXR) demonstrated consolidation. Antibiotic therapy with meropenem, vancomycin, and metronidazole was kept up; IVIIG and GCSF were added to the treatment. According to cardiac consult and echocardiography, dilated LV, LA, RV, pulmonary HTN, and left-sided pleural effusion were reported, and hence, Lasix and fluid restriction were ordered. On day seven, the neonate died due to cardiac arrest and multiorgan failure.

#### 3.4.3. Case 3

A 13-day-old full-term neonate, circumcised at eight days, was admitted. He was on antibiotic therapy and had a fever and poor feeding three days before hospitalization. Neonate's two-year-old sibling was deceased unexplainedly. On physical examination, he had depressed anterior fontanelle and spastic extremity. On day three, he experienced a seizure, and phenobarbital was started. Antibiotic therapy was preserved, and he underwent brain ultrasonography, which was not remarkable. Obtained EEG reported borderline epilepsy. Metabolic panel analysis suggested MSUD, confirmed with MRI; then, he was managed with MSUD-specific formula. After discovering leukopenia in the CBC test, a nasopharyngeal swab for COVID-19 was collected, which came positive. He was placed in isolation, and IVIG and GCSF were initiated. Consistently, his respiratory symptoms aggravated, and in spite of mechanical ventilation, he died after a while.

#### 3.4.4. Case 4

A premature male neonate was born through C-section and had bladder exstrophy. Due to ongoing repair surgery, he was transferred to NICU, and antibiotic therapy was initiated. After that, he presented with hematuria and bloody discharge from the tracheal tube and received fresh frozen plasma (FFP). During admission, he occasionally had difficulty in sucking and breathing; however, during the last five days, respiratory distress worsened, and he was intubated once more. While kidney failure, thrombocytopenia, raised LDH and CRP levels, and CXR alterations were presented, COVID-19 infection was suspected, and his RT-PCR test was positive. Furthermore, brain ultrasonography revealed IVH grade I on day 14. Owing to hyperkalemia and progressive sepsis, he died after 18 days.

#### 3.4.5. Case 5

A primigravida woman with hypothyroidism at 28 gestational weeks was admitted for an emergent cesarean section due to PROM and labor pain. A premature male infant was born with APGAR scores of 8/10. After ten minutes, his O_2_ saturation was dropped; hence, he was intubated immediately, transferred to NICU, and given surfactant therapy. He experienced a seizure episode on the second day characterized by jerking movements, and phenobarbital started. Because of sticky and viscous discharge from the tracheal catheter, the sample was collected for the RT-PCR test for COVID-19, which came positive. Laboratory evaluation showed anemia, hypoalbuminemia, leukopenia, and hyperglycemia. Eventually, his O_2_ saturation decreased to 45%, and he died despite support and resuscitation attempts.

## 4. Discussion

The present study is aimed at investigating the clinical characteristics and outcomes of 25 neonates confirmed with an RT-PCR test for COVID-19 in Fars Province, Iran, via a retrospective approach retrieving medical records from March 21, 2020, to March 20, 2021. The infection rate of neonates with COVID-19 is considered to be very low in many studies, and most reports have investigated maternal and neonatal outcomes of COVID-19 pregnant women. Therefore, this study is among the few reports on the incidence, clinical features, and outcomes of COVID-19-confirmed neonatal cases in Iran, which can shed a light on our understanding of the ambiguities in this regard [1, 13–17].

Limited data is available on the incidence rates of COVID-19 in neonates worldwide, possibly due to reporting larger age groups for better analysis. A systematic review found 1214 confirmed cases of COVID-19 in children under five, with 53% being infants and only 1% being newborns [5]. However, this study is outdated, and there is a lack of current knowledge about neonatal COVID-19 infection rates. Using a national survey, a recent preprint study, which claimed to be the largest study on neonatal COVID-19 infection in Iran, reported 825 positive PCR tests from February 2020 to February 2021 [18]. While data on incidence rates in different provinces of Iran were not specified, this study was generally reported to recruit cases from a national registry system. Meanwhile, chronologically, our study almost syncs with the time frame of this study; thus, it can be cautiously contemplated that the Fars Province has had 3.0% (25 out of 825) of the total neonatal COVID-19 cases in Iran.

In this study, regarding gender, male neonates accounted for the most confirmed cases of COVID-19 (60%). Interestingly, there are similar reports showing the same pattern of gender distribution; however, due to small sample sizes and other limiting factors, it is not logical to make any interpretation from this event unless further investigation is carried out [9, 19, 20].

The results of our study represent a fully symptomatic state of the neonates confirmed for COVID-19 infection. This could basically stem from the physician's perspective on available current evidence about the low infection rate among this age group, which may result in less frequent diagnostic testing requests unless some symptoms appear in suspected cases following national guidelines. Consistent with our findings, a retrospective cohort study on 19 neonates infected with COVID-19 from ten hospitals throughout Iran also reported the same pattern with the majority (89%) of the investigated cases being symptomatic [16]. From a different perspective, there is supportive evidence for asymptomatic COVID-19 infection in general in children as well as infants due to a more effective innate immune response, higher respiratory reserve, and less underlying disease [21–23]. Nevertheless, consistent with our findings, the majority of the studies report fever, respiratory and gastrointestinal symptoms, distress, and poor feeding among the manifested symptoms in symptomatic neonates with COVID-19 infection [9, 18, 24, 25].

Following the severe symptoms, NICU admissions occurred in 72% of our study cases, whereas another national retrospective Iranian study reported that 26% of the neonates required NICU admissions [16]. On the other hand, mechanical ventilation requirement was applied for only 24% of our cases, which is consistent with the findings of an epidemiological study on neonates with COVID-19 in Spain and another recent systematic review providing data in this regard [19, 22].

The majority (62.5%) of investigated neonates in the current study had a term gestation (≥37 weeks), which is similar to the previous findings in this regard [19, 26, 27]. In line with other studies, the median birth weight in our study (2900 g) was within the normal range (2500-4000 grams) [27, 28]. Also, 50% of the cases were fed formula milk, the rest were breastfed (45%), and one case was fed milk obtained from a milk bank. A recent study conducted in the United Kingdom showed no differences between postnatal COVID-19 infection in breastfed versus formula-fed infants [29]. Besides, another study did not detect the COVID-19 virus in the breastmilk of the infected mothers and recommended continuation of the feeding with preventive cautions regarding droplet transmissions [30].

Neonatal laboratory tests in this study, including WBC count, lymphocyte percentages, platelet count, and C-reactive protein, indicated that the majority of the cases were within the normal ranges, which confirms the findings of Al-Matary et al. [14]. However, other studies are reporting elevated WBC count, C-reactive protein, lymphopenia, and thrombocytopenia [18, 19, 31, 32].

Although determining the source of the infection is a difficult task, our study showed documented close contact with COVID-19 patients in around 60% of the neonates, while in 37% of neonates, their mothers had a confirmed COVID-19 infection. This is also in line with a recent systematic review showing 50% contact of neonates with infected mothers and the rest with other sources of infection (e.g., family members or health caregivers) [22]. Also, our results indicated that the source of COVID-19 is uncertain in almost half of the reported cases, which suggests that there may be gaps in our understanding of the transmission dynamics of the virus. In cases where the source of transmission is known, the results suggest that the virus is mainly transmitted through family, community, and vertical transmission. Overall, these results highlight the need for continued research into the transmission dynamics of SARS-CoV-2 to improve our understanding of the virus and inform effective public health policies aimed at controlling the spread of COVID-19. However, as of now, there is no solid proof indicating the occurrence of intrauterine transmission of SARS-CoV-2 [33]. It is also possible that neonates may acquire the virus through close contact with infected family members or healthcare workers [34].

The median age on admission in this study was four days with a range of directly after birth to 25 days old. A case series of 36 infants less than three months old who were infected with COVID-19 in Saudi Arabia reported 16.5 days of age as the median age on admission [35]. Meanwhile, the median hospitalization duration within the cases of our study was ten days, which is similar to the results of a systematic review study conducted by Trevisanuto et al. [22]. There are some other studies reporting much less hospitalization duration, with median of four days in neonates with COVID-19 [19, 36, 37]. Although the currently available literature emphasizes the worst prognosis with longer hospital stays in adults, there are ongoing debates on the likelihood of NICU admission in neonates [38].

To the best knowledge of the authors of this study, there are no rigorous scientific data on treatment plans for neonates with COVID-19 infection. This may arise from the low infection rate in this age group, and the attention has been mostly on pregnant women with COVID-19 and their neonatal outcomes in the follow-up [39]. There is no available evidence supporting the role of antiviral medications, corticosteroids, or immunoglobulins in the treatment of severe COVID-19 in neonates. In line with this debate, we observed the infrequent use of such medications in the treatment of neonates: 4% antiviral medications and 24% corticosteroids.

In this study, 20% of COVID-19-infected neonates died, which is a considerable rate compared to the available literature, mostly reporting much lower mortality rates. Trevisanuto et al. reported a 100% live discharge rate of neonates in their systematic review [22]. Also, UK databases have reported no deceased neonatal cases with a related cause to COVID-19 infection till April 2020 [15]. Meanwhile, the largest epidemiological study on 825 PCR-positive COVID-19 neonates in Iran reported a mortality rate of 10.3% with the need for respiratory support as the most important factor for neonatal death [18]. This is in line with our findings as all five deceased cases required respiratory support during hospitalization.

The current study had some potential limitations. Firstly, we only used the retrospective data and investigated the cases with positive RT-PCR tests for COVID-19 infection, and the other suspected cases were not included. Secondly, the small sample size of neonates confirmed with COVID-19 may limit the generalizability of the findings to other populations and restricted us to compare the characteristics of survived and deceased neonates. Aside from the notable false negative rate of RT-PCR testing for the COVID-19 infection, the source of attained specimens was not clear in the medical recordings, which we used. Another limitation of this study was the unclearness of the direct effect of COVID-19 as the main reason for death in the deceased cases while they already had other ongoing clinical problems as well. However, this is the first study in Iran reporting an incidence of COVID-19 infection in neonates born in Fars from March 2020 to March 2021. Besides, we followed each case during the neonatal period and reported their outcomes. This study highlights the significant burden of COVID-19 infection among neonates in Fars Province, with a high neonatal mortality rate. The findings provide valuable insights into the clinical and treatment-related characteristics of infected neonates and contribute to a better understanding of the impact of the pandemic on neonatal healthcare. Despite the limitations, these results underscore the need for continued efforts to prevent and manage COVID-19 infection in this vulnerable population.

## 5. Conclusion

This study revealed the incidence rate of confirmed and symptomatic COVID-19 infection among neonates born in Fars, one of the largest provinces in Iran considering 47.5 : 100000 living births. While in most studies, newborns were a term with appropriate birth weight, the neonatal mortality rate was 20%. Despite the limitations, the study adds to the currently available literature by providing informative data on clinical, paraclinical, and treatment-related characteristics of infected neonates with COVID-19, which can shed a light on a better understanding of the effects of the pandemic on neonatal healthcare along with giving a regional snapshot regarding the management of COVID-19 infection in these neonates.

## Figures and Tables

**Table 1:** Demographic and medical history of neonates with confirmed COVID-19 infection.

Characteristics of the neonates	Infant outcome		
	Total (*N* = 25)	Recovered (20, 80%)	Deceased (5, 20%)
Birth weight (grams)	2900 (1490-3770)	2900 (1490-3770)	2900 (1490-3670)
Birth height (centimeters)	50 (33-76)	50 (33-56)	48 (33-76)
APGAR score (minute 1)	9 (7-9)	9 (7-9)	9 (8-9)
APGAR score (minute 5)	10 (8-10)	10 (8-10)	10 (10-10)
Gender			
Male	15 (60%)	13 (86%)	2 (13%)
Female	10 (40%)	7 (70%)	3 (30%)
Gestational age at birth			
≥37 weeks	15 (60%)	12 (80%)	3 (20%)
<37 weeks	9 (36%)	7 (77.8%)	2 (22.2%)
Multifetal pregnancy			
Yes	2 (8%)	2 (100%)	0 (0%)
No	23 (92%)	18 (78.3%)	5 (21.7%)
Neonate's blood group			
A	4 (16%)	4 (100%)	0 (0%)
B	5 (20%)	5 (100%)	0 (0%)
AB	2 (8%)	1 (50%)	1 (50%)
O	9 (36%)	6 (66.7%)	3 (33.3%)
Rh			
Positive	19 (76%)	16 (84.2%)	3 (15.8%)
Negative	1 (4%)	0 (0%)	1 (100%)
Feeding type			
Breastfeeding	11 (44%)	10 (90.9%)	1 (9.1%)
Formula milk	12 (48%)	8 (66.7%)	4 (33.3%)
Milk bank	1 (4%)	1 (100%)	0 (0%)
Congenital anomalies/metabolic diseases			
Yes	10 (40%)	6 (60%)	4 (40%)
No	15 (60%)	14 (93.3%)	1 (6.7%)

Data were reported as median (minimum-maximum) or frequency (%) as indicated. Abbreviations: APGAR: appearance, pulse, grimace, activity, and respiration.

**Table 2:** Clinical and paraclinical features of infants with confirmed COVID-19 infection.

Characteristics of the neonates	Infant outcome		
	Total (*N* = 25)	Recovered (20, 80%)	Deceased (5, 20%)
Postnatal age at admission (days)	4 (0-25)	2 (0-25)	12 (10-27)
Duration between symptom onset to detection (days)	2 (0-14)	2 (0-14)	2 (1-14)
Hospitalization duration (days)	10 (1-29)	10.5 (1-29)	9 (3-19)
Neonatal clinical symptoms			
Fever			
Yes	12 (48%)	10 (83.4%)	2 (16.6%)
No	13 (52%)	10 (76.9%)	3 (23.1%)
Cough			
Yes	2 (8%)	2 (100%)	0 (0%)
No	23 (92%)	18 (78.3%)	5 (21.7%)
Respiratory distress			
Yes	9 (64%)	5 (55.6%)	4 (44.4%)
No	16 (36%)	15 (93.7%)	1 (6.3%)
Lethargy			
Yes	12 (48%)	9 (75%)	3 (25%)
No	13 (52%)	11 (84.6%)	2 (15.4%)
Poor feeding			
Yes	13 (52%)	10 (76.9%)	3 (23.1%)
No	12 (48%)	10 (83.3%)	2 (16.7%)
Gastroenteritis			
Yes	3 (12%)	3 (100%)	0 (0%)
No	22 (88%)	17 (77.3%)	5 (22.7%)
Asymptomatic			
Yes	0	0 (0%)	0 (0%)
No	25 (100%)	20 (80%)	5 (20%)
Neonatal laboratory tests			
C-reactive protein			
Positive	2 (8%)	1 (50%)	1 (50%)
Negative	20 (80%)	18 (90%)	2 (10%)
White blood cell count (cells/*μ*L)			
<4000	2 (8%)	1 (50%)	1 (50%)
4000-12000	13 (52%)	11 (84.6%)	2 (15.4%)
>12000	7 (28%)	7 (100%)	0 (0%)
Lymphocyte (%)			
<20%	1 (4%)	0 (0%)	1 (100%)
20-40%	5 (20%)	5 (100%)	0 (0%)
>40%	6 (24%)	6 (100%)	0 (0%)
Neutrophil (%)			
<20%	2 (8%)	2 (100%)	0 (0%)
20-40%	3 (12%)	3 (100%)	0 (0%)
>40%	7 (28%)	6 (85.7%)	1 (14.3%)
Platelets (×10^3^/*μ*L)			
<150	3 (12%)	2 (66.7%)	1 (33.3%)
150-450	16 (64%)	15 (93.7%)	1 (6.3%)
>450	3 (12%)	2 (66.7%)	1 (33.3%)
Close contact COVID-19			
Yes	13 (52%)	12 (92.3%)	1 (7.7%)
No	9 (36%)	5 (55.6%)	4 (44.4%)
Family history of COVID-19			
Yes	9 (369%)	8 (88.9%)	1 (11.1%)
No	12 (48%)	9 (75%)	3 (25%)
NICU admission			
Yes	18 (72%)	13 (72.2%)	5 (27.8%)
No	7 (28%)	7 (100%)	0 (0%)
Mechanical ventilation			
Yes	6 (24%)	3 (50%)	3 (50%)
No	19 (76%)	17 (89.4%)	2 (10.6%)
Antibiotic therapy			
Yes	25 (100%)	20 (80%)	5 (20%)
No	0 (0%)	0 (0%)	0 (0%)
Corticosteroids			
Yes	6 (24%)	4 (66.7%)	2 (33.3%)
No	19 (76%)	16 (84.2%)	3 (15.8%)
Antiviral medication			
Yes	1 (4%)	0 (0%)	1 (100%)
No	24 (96%)	0 (0%)	0 (0%)

**Table 3:** Maternal risk factors for neonatal COVID-19 infection.

Characteristics of the mothers	Infant outcome		
	Total (*N* = 25)	Recovered (20, 80%)	Deceased (5, 20%)
Maternal age (years)			
High risk (<19 or>35)	5 (20%)	5 (100%)	0 (0%)
Low risk (19-35)	20 (80%)	15 (75%)	5 (25%)
Comorbid conditions in mothers			
Diabetes mellitus (DM)	3 (12%)	2 (66.7%)	1 (33.3%)
Chronic hypertension	1 (4%)	1 (100%)	0 (0%)
Hypothyroidism	6 (24%)	5 (83.3%)	1 (16.7%)
Epilepsy	1 (4%)	0 (0%)	1 (100%)
Addiction	2 (8%)	2 (100%)	0 (0%)
Maternal fever on admission			
Yes	5 (20%)	4 (80%)	1 (20%)
No	20 (80%)	16 (80%)	4 (20%)
Premature rupture of membranes			
Yes	2 (8%)	1 (50%)	1 (50%)
No	23 (92%)	19 (82.6%)	4 (17.4%)
Mode of delivery			
Vaginal delivery	10 (40%)	6 (60%)	4 (40%)
Cesarean section	15 (60%)	14 (93.3%)	1 (6.7%)
C/S indication			
Previous C/S or voluntary	7 (46%)	7 (100%)	0 (0%)
Comorbidity	3 (20%)	2 (66%)	1 (33%)
Uncertain	5 (33%)	5 (33%)	0 (0%)
COVID-19 PCR test			
Positive	9 (36%)	8 (88.9%)	1 (11.1%)
Negative	13 (52%)	9 (69.2%)	4 (30.8%)
Not tested	2 (8%)	2 (100%)	0 (0%)
Mother-infant isolation			
Yes	8 (32%)	6 (75%)	2 (25%)
No	16 (64%)	13 (81.2%)	3 (18.8%)

## Data Availability

Data is available on request from the corresponding author.
